# Unveiling a Ghost Proteome in the Glioblastoma Non-Coding RNAs

**DOI:** 10.3389/fcell.2021.703583

**Published:** 2021-12-23

**Authors:** Tristan Cardon, Isabelle Fournier, Michel Salzet

**Affiliations:** ^1^ University of Lille, Inserm, CHU Lille, U1192—Protéomique Réponse Inflammatoire Spectrométrie de Masse—PRISM, Lille, France; ^2^ Institut Universitaire de France, Paris, France

**Keywords:** alternative proteins, glioblastoma, LncRNA—long noncoding RNA, SEPs, ncRNA (noncoding RNA), brain cancer, mass spectrometry—LC-MS/MS

## Abstract

Glioblastoma is the most common brain cancer in adults. Nevertheless, the median survival time is 15 months, if treated with at least a near total resection and followed by radiotherapy in association with temozolomide. In glioblastoma (GBM), variations of non-coding ribonucleic acid (ncRNA) expression have been demonstrated in tumor processes, especially in the regulation of major signaling pathways. Moreover, many ncRNAs present in their sequences an Open Reading Frame (ORF) allowing their translations into proteins, so-called alternative proteins (AltProt) and constituting the “ghost proteome.” This neglected world in GBM has been shown to be implicated in protein–protein interaction (PPI) with reference proteins (RefProt) reflecting involvement in signaling pathways linked to cellular mobility and transfer RNA regulation. More recently, clinical studies have revealed that AltProt is also involved in the patient’s survival and bad prognosis. We thus propose to review the ncRNAs involved in GBM and highlight their function in the disease.

## Introduction

With 80% of the lethal brain cancer in USA with an incidence of 3.21/100.000, glioblastoma (GBM) is the main malignant primary brain tumor (Paulmurugan et al., 2019). The prognosis of GBM remains poor with a median survival of about 15–16 months (Weller et al., 2017) for a median age at diagnostis of 52 years and only about 5% patients surviving more than 5 years (Ostrom et al., 2018). The GBM standard treatment is based on the resection of the tumor at best when possible followed by combined radiotherapy and chemotherapy with temozolomide.

GBM classification is intrinsincly difficult due to the tumor heterogeneity. This has led to an evolution of the World Health Organization (WHO) classification from a classification principally based on histological criteria (Louis et al., 2007) to a refined classification integrating molecular data and based on the absence/presence of isocitrate dehydrogenase (IDH) mutation and 1p/19q mutation edited in 2016 (Louis et al., 2016). Following the new classification which combines both histological criteria and molecular features, three groups of tumors are described: 1) the IDH wild-type group which represents 90% of the GBM and has for morphological specificity the presence of giant cells, gliosarcoma, and epithelioid GBM, 2) the IDH mutated group which represents less than 10% of GBM, and 3) the GBMs whose IDH status is unknown and that are referred as not otherwise specified (NOS). For glioma tumors with more than one genetic determinant such as the IDH mutant and 1p/19q-codeleted, these are classified as oligodendroglia, and the IDH mutants which are 1p/19q non-codeleted fall in the astrocytic class. In 2018, new molecular features have been described including epidermal growth factor receptor (EGFR) amplification, losses of chromosome 10, gains of chromosome 7, and telomerase reverse transcriptase (TERT) promoter mutations to better classify low-grade diffuse astrocytic glioma (WHO grade II or III neoplasm) with evolution close to GBM (Brat et al., 2018; Wesseling and Capper, 2018).

Overall, many efforts have been made to increase the number of biomarkers and their specificity, thus allowing for a rapid and more specific diagnosis. GBM subclassification is largely based on genomic and transcriptomic data. Already in 2008, specific genetic alterations were reported including the p53, RB, RTK, RAS, and PI3K proteins (Brennan et al., 2013). In 2010, four subtypes of GBM (proneural, neural, classic, and mesenchymal) have been categorized based on the transcriptomic analysis; however, it was shown to be less homogenous than the genomic one (Verhaak et al., 2010; Wang et al., 2017). More recently, integrated pharmaco-proteogenomic studies were performed on GBM resulting in two subgroups within the IDH wild-type group showing different prognostic and therapeutic opportunities (Oh et al., 2020). Moreover, the integrative analysis of the protein profile, the RNA expression, and the patient clinical information enable the identification of specific signaling pathways related to immune, metabolic, and developmental processes and associated with a patient survival (Yanovich-Arad et al., 2021). Recently, subclassification of GBM using MALDI mass spectrometry imaging associated with spatially resolved proteomics and patient survival has established the presence of three GBM subtypes corresponding to antiviral immune response, neurogenesis processes, and immune infiltration. Ten prognostic markers were identified among which one is a protein issued from an ncRNA (Drelich et al., 2020).

Most of the diagnostic and prognostic markers have been searched and identified in the conventional coding molecular landscape of RNA and corresponding unique proteins. However, if approximately 90% of the genome is transcribed into RNA, the Encyclopedia of DNA Elements (ENCODE) project has shown that only 25% is actively transcribed into mRNA (Djebali et al., 2012; Dunham et al., 2012). In addition, only 2% of these RNA would be translated into known proteins. Thus, a large part (75%) of the genes codes for noncoding RNA transcripts (ncRNA), which represent the hidden part of the transcriptome (DeOcesano-Pereira et al., 2020). Very interestingly, 40% of the ncRNAs are expressed in the brain (Latowska et al., 2020). In the case of GBM, ncRNAs have been found to be involved in many processes related to cancer such as stemness/differentiation, proliferation, invasion, survival, DNA damage response, and chromatin dynamics, and they are thus considered as promising new therapeutic and diagnostic markers (Stackhouse et al., 2020). The study of ncRNAs has revealed a coding dimension, despite its designation as “noncoding” RNA. Indeed, uncharacterized and unreferenced proteins issued from ncRNA have recently been identified. Despite that various terminologies have been employed to describe these proteins such as small or short proteins (smProt), sORF-encoded peptides (SEPs, sPEP), alternative proteins (AltProt), and micropeptides (miPEPs), they all share the same characteristics, i.e., they harbor an unreferenced and unpredicted open reading frame (ORF) on a nucleic acid sequence previously considered as noncoding. The finding of these novel proteins has opened the door of the so-called ghost proteome (Cardon et al., 2021a). Thus, beyond the conventional proteins, a large set of hidden proteins issued from the nc RNAs or noncoding regions of mature RNAs (mRNAs) exist, which demonstrate a strong prognostic and diagnostic value. Thus, for GBM, this ghost proteome represents an opportunity for new diagnostic targets and a second generation of therapeutics in a high therapeutically demanding pathology. Taken together, this review will cover the latest molecular discoveries on ncRNAs and their issued AltProts to shed light on the dark molecular landscape associated with GBM pathology.

## The Non Coding RNA

### Brief Insight Into ncRNA

**FIGURE 1:**
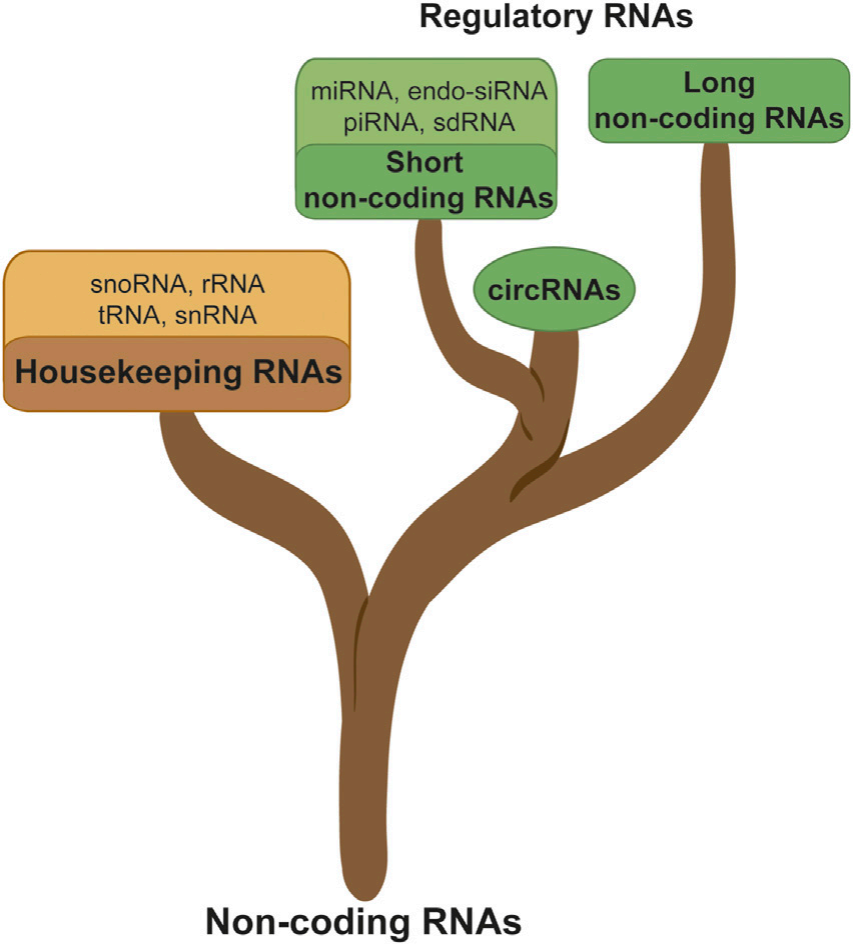
Noncoding RNA classification; ncRNAs are subdivided into different branches such as the housekeeping or regulatory RNA, sncRNA, lncRNA, and circRNA.

The ncRNAs are referring to the RNAs which were assumed not to be translated into proteins. ncRNAs have been shown to harbor important cellular functions including some which are essential to maintaining the basic cellular functions. The housekeeping RNAs include the small nucleolar RNAs (snoRNA), small nuclear RNAs (snRNA), transfer RNAs (tRNA), ribosomal RNAs (rRNA), and regulatory RNAs. The regulatory RNAs are the RNA family which is the most involved in cancer progression. It is subcategorized into three main groups, namely, the short non-coding RNAs (sncRNA, <200 nucleotides), the long non-coding RNAs (lncRNA, >200 nucleotides), and the circular RNAs (circRNA). The sncRNAs are further divided into microRNAs (miRNA), endogenous short interfering RNAs (endo-siRNA), Piwi-interacting RNAs (piRNA), and self-deliverable siRNAs (sdRNA). LncRNAs are characterized by their length (>200 nucleotides) and are classified based on their locus position on the genome such as sense, antisense, bidirectional, intronic, intergenic, or enhancer (Latowska et al., 2020), as shown in Figure 1.

### SncRNA

The sncRNAs do not contribute to translation due to their very small size unlike lncRNAs and circRNAs which are much more suitable matrices for protein translation (Figure 1). However, sncRNAs are important components in the oncogene and tumor suppressor network and were considered as potential biomarkers, therapeutic reagents, and therapeutic targets for cancer treatment (Wang et al., 2019). However, among this class of RNAs, we find miRNAs. miRNAs are a class of RNAs described to be involved at several levels in the regulation of tumors and in particular in glioma. If they are too short to be translated, their sequence was described to interact with 3′UTR regions of mRNA having the effect of inhibiting their translation. This phenomenon can have consequences such as tumor suppression (e.g., miR-7, miR-34a, miR128) but also the stimulation of oncogenesis (e.g., miR-10b, miR-21, miR-93), and finally many miRNAs occur, in signaling pathways linked to cancerization: apoptosis, angiogenesis, drug resistance (Chen et al., 2021). This interaction can also inhibit the expression of the AltProt expression from the 3′UTR and other regions from the mRNA targeted by the miRNA, a non-explored aspect of the miRNA function. Nonetheless, miRNAs have been also described to be translated based on their first structure, the primary miRNA (pre-miRNA). The miRNA-encoded peptides (miPEPs) are encoded from pre-miRNAs and are known to be involved in cancer. For example, miPEP133 is expressed in the normal human colon, stomach, ovary, uterus, and pharynx (Kang et al., 2020). miPEP-200a and miPEP-200b inhibit the migration of prostate cancer cells by regulating the epithelial-to-mesenchymal transition (EMT) of these cells (Wang et al., 2019). Nevertheless, none of these miPEPs have yet been identified in GBM.

### Function of lncRNA

**FIGURE 2:**
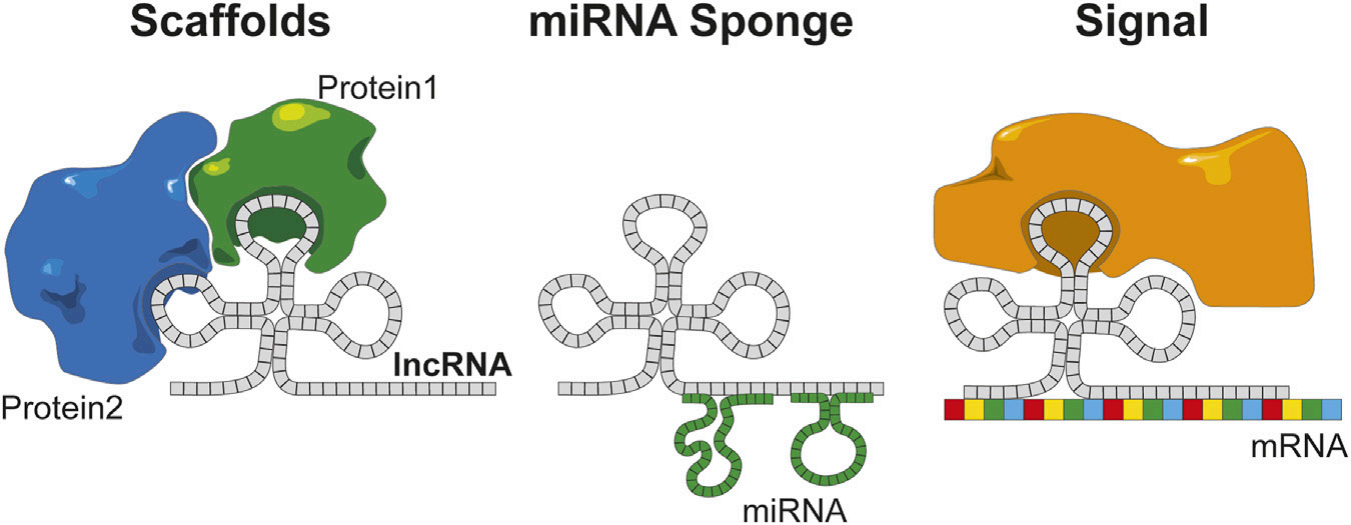
lncRNAs have several functions based on the structural organization of the sequences. Scaffold of protein maintains protein interaction, miRNA sponge regulates and inhibits the miRNA on the cell, and signal combines an RNA to a protein that can trigger the translation.

LncRNAs bear close characteristics to mRNAs such as the presence of a 7-methyl guanosine 5′ cap and for many of them a poly-adenylated tail, which is why some can be detected by standard poly-A sequencing assays. Initially, given the translation rules that have been established, they were not predicted to translate into proteins. However, lncRNAs do present ORFs and methods for monitoring the ribosome binding have shown that many lncRNAs are translated. The abundance of lncRNAs in the cell is estimated to be 10 times lower than those of encoding genes; nevertheless, it is interesting to note that lncRNAs are generated through a similar translation mechanism to mRNAs (Dunham et al., 2012). This confirms the interest of lncRNAs in search for new biomarkers and therapeutic targets. LncRNAs have been shown to be involved at different levels of signaling pathways, and their variation are related to cellular dysregulation involved in pathophysiological mecahnisms. For example, lncRNAs can regulate gene expression through the fixation to DNA, although activity at the transcriptomic level has also been found. Indeed, through the fixation to mRNA, lncRNAs can inhibit protein translation. Finally, at the proteomic level, they can interact with the active sites of enzymes or proteins to modulate their activities thanks to their hairpin structure (Stackhouse et al., 2020). At the present time, the function of only few lncRNAs has been experimentally validated (Fang and Fullwood, 2016), but there is an exponentially growing interest for this peculiar RNA category. LncRNAs are divided into three functional categories according to their mode of interaction (Figure 2) (Stackhouse et al., 2020). When their function depends on their structure, they act as “scaffolds,” e.g., during an interaction with proteins or enzymatic inhibition/activation. When they fix to a factor (miRNA, transcription factor, etc.) to reduce the number of copies available in the cell, they act as a “sponge.” Lastly, when lncRNAs bridge the gap between an enzyme/protein or an RNA/DNA allowing the initiation/repression of the translation depending on the target, they will serve as “signals” (Latowska et al., 2020). Moreover, in a pathophysiological process such as cancer, a direct correlation between the expressions of the coding gene and lncRNAs has been observed. Among the different signaling pathways found, the most represented were related to cell proliferation, senescence, apoptosis, and dysregulation of the cellular process, all well-known cancer hallmarks.

### Implication of lncRNA in GBM

In GBM, lncRNAs are considered as putative therapeutic targets, due to the significant variation in their expression in the pathology and its correlation with cancer types (Paulmurugan et al., 2019). A pioneering study was carried out using data from The Cancer Genome Atlas (TCGA) (Tomczak et al., 2015) and led to the identification of variation in the expression of 1,995 mRNAs and 398 lncRNAs, among which 98 have been then experimentally tested and were shown to be involved in functions related to development, metastasis formation, and tumorigenesis (Li et al., 2016). While large-scale studies have enabled the identification of large numbers of lncRNAs linked to a specific physiopathological environment, targeted studies of specific lncRNAs offer an in-depth exploration of the mechanisms and functions of these lncRNAs in a given pathology. Among the lncRNAs which have been subjected to dedicated studies, HOTAIR, ANRIL, and PARTICLE lncRNAs have been found to play a role in chromatin remodeling, thus influencing epigenetics. PANDA, RMST, and HOXA1 lncRNAs regulate the translation mechanisms of mRNAs while HMMR-AS1 lncRNA is involved in the stabilization of mRNA and therefore protein expression (Latowska et al., 2020). Others such as MALAT1, LBX2-AS1, ST7-AS1, and DDX11-AS1 play a role in cell proliferation, migration, and tumor invasion, and some were associated with patient survival (OBI1-AS1). In the following section, a detailed presentation of these lncRNAs is given.

HOTAIR–HOX antisense intergenic RNA has been identified in a study dedicated to the characterization of the transcriptional landscape of the four *HOX* loci and their role in the developmental epigenetics (Rinn et al., 2007). HOTAIR is a 2,158-nucleotide poly-adenylated lncRNA with six exons (Hajjari and Salavaty, 2015) which is derived from the antisense transcription of the *HoxC* gene on chromosome 12q13.13. HOTAIR is involved in chromatin remodeling and has been shown to correlate with the survival of patients with GBM (Paulmurugan et al., 2019). Moreover, the expression of HOTAIR in the serum of GBM patients has shown a value for the diagnosis and prognosis of the GBM severity (Stackhouse et al., 2020).

MALAT1—the metastasis-associated lung adenocarcinoma transcript 1, also known as noncoding nuclear-enriched abundant transcript 2 (NEAT2), was described for the first time in 2003 (Ji et al., 2003). MALAT1 is an lncRNA of more than 8,000 nucleotides encoded from chromosome 11q13. It has been identified by subtractive hybridization to be differentially expressed at different stages of non-small lung cancer and to be associated with metastasis formation (Gutschner et al., 2013). A correlation was also found between the cellular expression of MALAT1 in non-small lung cancer (NSCLC) (Ji et al., 2003) and the presence of metastasis in the brain (DeOcesano-Pereira et al., 2020). In GBM, the regulation of MALAT1 by NF-κB and p53 was identified during temozolomide treatment. MALAT1 inhibits temozolomide activity, and combining the silencing of MALAT1 by siRNA to temozolomide has revealed to increase the treatment efficiency (Voce et al., 2019). More recently, MALAT1 has been correlated with the expression of miR-124 and an increased expression of the ZEB2 gene, which regulates the cell proliferation and tumor progression in GBM (Cheng et al., 2021).

HMMR-AS1—hyaluronan-mediated motility receptor antisense RNA1 (also called RP11-80G.1) is an lncRNA antisense encoded on chromosome 5p34. It was first described in 2018 in breast cancer as a regulator of cell proliferation and migration, thus influencing tumor progression (Liu et al., 2016). More recently, HMMR-AS1 has been identified as overexpressed in GBM. The inhibition of HMMR-AS1 reduces the expression of HMMR and results in the suppression of tumor growth (Li et al., 2018).

ST7-AS1—suppression of tumorigenicity 7 antisense RNA 1 which is derived from the reverse transcription of the chromosome region 7q31.2 is found to be underexpressed in the tissues of patients with GBM. ST7-AS1 binds and inhibits polypyrimidine tract-binding protein 1 (PTBP1), thus suppressing the Wnt/β-catenin signaling and leading to an increase in the tumor progression (Sheng et al., 2021).

DDX11-AS1—DEAD box 11 antisense RNA1 (also known as ATP-dependent DNA helicase DDX11) is transcribed from a gene located on chromosome 12p11.21 which has been evidenced in a study on hepatocellular carcinoma in 2017 (Shi et al., 2017). DDX11-AS1 was shown to be an oncogene upregulated in many cancers (colorectal, osteosarcoma, bladder, gastric, and NSCLC) which was also recently found to be implicated in GBM (Feng et al., 2020). In GBM, DDX11-AS1 is upregulated and associated with a poor survival prognosis. It is involved in several signaling pathways such as tumor cell proliferation, migration, and invasion. It acts as an miRNA sponge, targeting in particular miR-499b-5p which is involved in the regulation of the expression of the GBM oncogene RWDD4 (Zheng et al., 2021).

**LBX2-AS1**—ladybird homeobox two antisense RNA1, which is transcribed from chromosome 2p13.1, is associated with GBM. It regulates the Akt/GSK3b pathway, therefore impacting the proliferation, migration and invasion of glioma cells (Wen et al., 2021).

**OBI1-AS1**—ORC ubiquitin ligase 1 antisense RNA 1, also known as **POU4F1-AS1**—POU class 4 homeobox 1 antisense RNA 1 (*referenced under* ***RNF219-AS1***), is translated from chromosome 13q22.3. Preliminary data have shown that this lncRNA is downregulated in GBM and linked to poor overall survival. Function prediction based on gene ontology and signaling pathway enrichment indicates a potential role in pluripotency and oligodendrocyte differentiation (Mamivand et al., 2021).

In summary, the transcriptomic landscape analysis has shown to be much more complex than previously expected with many promising lncRNAs being expected to serve as future biomarkers and therapeutic targets. However, the function of all these lncRNAs and the signaling pathways they are acting in remain yet to be discovered. Moreover, the recent discovery of proteins translated from ncRNAs is adding a level of complexity that needs to be addressed as well.

## A Ghost Proteome Is Hiding Behind Non Coding RNA

### LncRNA-Encoded Proteins

Historically, protein translation from mRNA can be predicted following well-established and well-described rules where the ribosome binds and reads the mRNA sequence to translate nucleic acids into an amino acid sequence. Nevertheless, thanks to ribosome profiling (Ribo-seq), sequencing of translating mRNA (RNC-seq), and proteomics, several ncRNAs were identified in the last decade to translate polypeptides (Menschaert et al., 2013; Ingolia, 2014; Slavoff et al., 2014; Pueyo et al., 2016; Delcourt et al., 2018; Brunet et al., 2019; Cardon et al., 2020a). Among these lncRNA-encoded peptides, some are found in pathologies such as cancer. In breast cancer, Linc00908 translates the protein SMIM30 (also called ASRSP) (Wang et al., 2020) and CASIMO1 encodes the protein SMIM22 (Polycarpou-Schwarz et al., 2018), whereas in colon cancer, Loc90024 encodes the SRSP protein (Meng et al., 2020) and HOXB-AS3 translates the homonym protein (Huang et al., 2017). In lung cancer, Linc01420 was found to encode the NoBody protein (D’Lima et al., 2017) and UBAP1-AST6 the homonym protein (Ye et al., 2020). If the role of these proteins is not yet fully understood and they are not necessarily referenced in the protein databases, some have been accepted, such as NoBody (non-annotated P-body-dissociating polypeptide) which is referenced and considered as reviewed in the UniprotKB database since March 2017 (accession number A0A0U1RRE5, NBY). NoBody (NBY) is involved in the RNA decapping through its interaction with the protein enhancer of decapping 4 (EDC4), an activator of RNA decapping (D’Lima et al., 2017).

**FIGURE 3:**
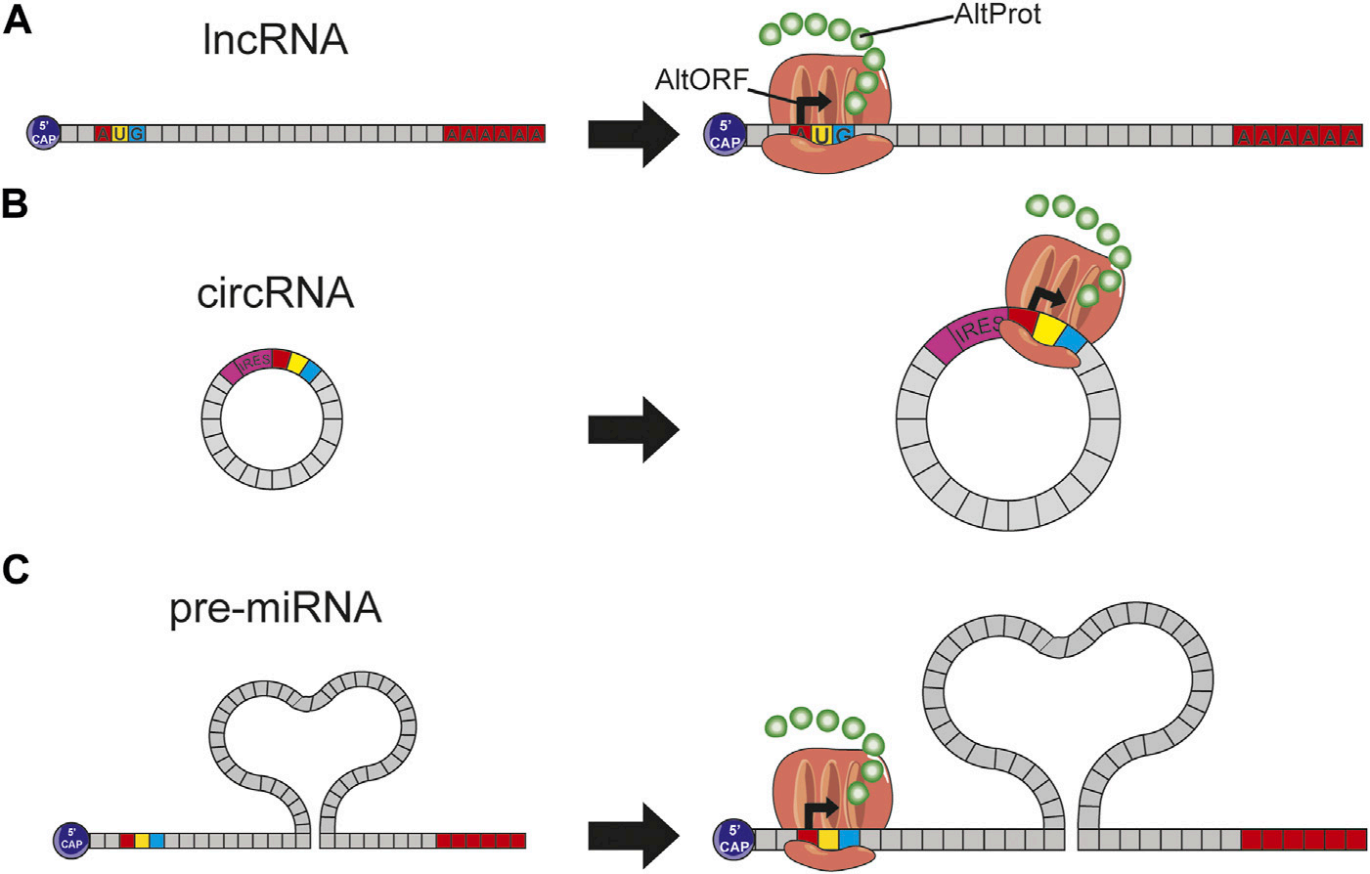
**(A)** The presence of a start codon on an lncRNA induces this translation into protein by the ribosome; this protein, not predicted in conventional databases, is therefore considered to be AltProt. **(B)** circRNA arbors an IRES site helping the ribosome fixation and thus the expression of some proteins. **(C)** Pre-miRNAs have on their sequence some start codons involving the ribosome binding and thus the transcription of AltProt.

Recent studies have demonstrated that a significant part of the proteome issued from ncRNAs or the noncoding part of mRNAs is missing. Nowadays, part of this hidden proteome has been uncovered and starts to be integrated into the reference protein databases, although they only represent a tiny portion by comparison to the thousand proteins predicted from lncRNA which still remain to be studied (Figure 3).

### A Historical Gap in the Dogma of Protein Translation

It was for long admitted that the DNA is transcribed into a pre-mature RNA (pre-mRNA) and then into several mature RNAs (mRNAs) through alternative splicing, each of these mRNAs being then translated into a single protein. However, modern MS-based proteomics thanks to the high sensitivity and speed of the new instruments has challenged the bases of molecular biology and translation (Mouilleron et al., 2016). Indeed, the protein databases were largely established by translation prediction from the first genome sequencing data. Rules have thus been established to determine which transcripts code for proteins and predict protein sequences. Among others, these rules establish that an RNA which is presenting an ORF of less than 100 nucleotides is considered as non-coding. Moreover, for the translation to occur, the presence of a start “AUG” codon as well as a Kozak context is required to enable the fixation of the ribosome (Kozak, 1999). Besides, it was considered that eukaryotic cells are monocistronic, i.e., that only a single protein can be translated from a specific coding mRNA (Breuza et al., 2016). However, contrary to this dogma, the development of new technologies of RNA sequencing such as polysome profiling, ribosome immunoprecipitation, ribosome affinity purification, ribosome profiling (also known as Ribo-seq), and ribosome-nascent chain complex sequencing (RNC-seq) and MS-based proteomics using databases built from new prediction rules (i.e., Open Proteome database) allowed the detection and identification of translated proteins from supposedly ncRNAs or non-coding regions of mRNA. The different studies have revealed that numerous ncRNAs were targeted by the ribosome and translated into proteins unlike what was initially expected (Menschaert et al., 2013; Bazzini et al., 2014; Ingolia et al., 2014; Pueyo et al., 2016; Wang et al., 2019). For a few years now, Ribo-seq is democratizing thanks to the development of one-shot analysis methods such as AHARIBO (Minati et al., 2021) which enables to find proteins derived from RNA so far considered as noncoding. Despite important advances, the implementation of proteogenomic strategies remains and requires a set of skills in the various fields of genomic, transcriptomic, and proteomic and access to often expensive technologies. However, a growing number of research are now focused on these forgotten proteins, many of them being focused on better prediction and the creation of new databases to promote the identification of this “ghost proteome” by MS-based large-scale proteomics (Cardon et al., 2021a).

### Filling the Gap

Demonstrating the existence of proteins encoded from ncRNA has highlighted the limits of the conventional protein databases and the need for new protein libraries (Wu et al., 2020). In the last 10 years, several new databases have thus been created. Many of them are based on the identification of small ORFs (smORF) in the ncRNAs to integrate these unreferenced sequences, at the protein but also at the genomic level, in new repositories, namely, sORF.org (Olexiouk et al., 2017), SmProt (Hao et al., 2018), and RPFdb (Xie et al., 2016). On the other hand, some databases are established based on the prediction of the translation sites and the smORF from genomic and transcriptomic data (RefSeq (O’Leary et al., 2016) and ENSEMBL assembly (Aken et al., 2017)) followed by a bioinformatic assembly, close to that initially carried out for the reference proteins (RefProt). These databases give access to new isoforms as well as to the proteins translated from noncoding sequences which are coined the terminology of alternative proteins (AltProt). The constitution of this public database, initially called HAltORF (2012) (Vanderperre et al., 2012) and now known as OpenProt (2019) (Brunet et al., 2019), was based on different translation rules than that of the classic RefProt databases. The used translation rules are as follows: no maximum length for the translated proteins, a minimum length transcript of 30 codons, and the use of an AUG initiation codon, allowing the generation of a larger number of predicted proteins. In addition, this database crosses the predicted proteins with the known data in Ribo-seq and MS allowing to combine a maximum of predictions for exploratory studies or to use a more restricted database only including proteins already described in the literature. OpenProt has been updated once more in 2021 (Brunet et al., 2021) to expand the information provided to the genome annotation with the inclusion of a browser to search for specific genes and their corresponding AltProts, the integration of the number of unique peptides found in the reanalysis of the MS data, and the identification of coding sequences presenting similarities with these genes. OpenProt is a major tool for the identification of novel proteins, particularly those derived from ncRNAs.

## The Ghost Proteome in the GBM

As previously described, ncRNAs have unexpected translational capacities. ncRNA-encoded proteins are potential new biomarkers and therapeutic targets in different cancers. Very interestingly, in GBM, the actual ghost proteome discovery was largely related to circRNAs.

### A Brief Insight Into circRNAs

Circular transcripts harbor the distinctive characteristic of forming a loop through an end-to-end junction, which links their 3′ terminal part to their 5′ end. This configuration prevents the 3′ polyadenylation as well as the 5′ capping (Greene et al., 2017). This architecture gives circRNAs a greater stability than linear RNAs because of the limited degradation, hence resulting in a larger abundance of circRNAs in cells. If their observation is possible, their function remains largely unknown. In addition to their relatively recent discovery, circRNAs are difficult to analyze due to their structure. However, studies focused on these circRNAs have shown that their functions could be linked to the inhibition of miRNA (sponge), the interaction of RNA-binding proteins, and the regulation of the expression of genes and transcripts. They have been shown in the regulation of signaling pathways related to tumorization, such as cell proliferation, invasion, and migration, which are cancer hallmarks. In general, circRNAs present a decreased abundance in tumor tissues compared to healthy ones (Latowska et al., 2020). In addition to the current knowledge on the function of circRNAs, some of them exhibit ORFs and have the ability to translate proteins. Recent studies have demonstrated the capacity of the ribosome to bind to RNA sequences and in particular circRNAs, independently of the presence of a 5′ cap. This internal ribosome entry site (IRES) element allows the direct binding of the subunit 40S ribosomal in synergy with the IRES transacting factor (ITAF) to initiate translation (Zhang et al., 2018a). Other translation mechanisms for circRNAs are possible such as the initiation of translation through the methylation of the adenosine base at the nitrogen-6 position (m6A) on the RNA. The m6A modification is observed on RNAs in specific contexts when cells are subjected to stress, during development, apoptosis, or the regulation of the cell cycle. This suggests that the proteins issued from these mechanisms are involved in the regulation of these processes (Kong et al., 2020) (see Figure 1).

### CircRNA-Encoded Alternative Proteins

Recently, different studies have reported circRNA-translated proteins (Zhang et al., 2018b; Wang et al., 2019; Xia et al., 2019; Wu et al., 2020). In GBM, the expression of these AltProts translated from circRNAs has been correlated with the development of the pathology (Kong et al., 2020). In 2018, in one of the first studies experimentally describing the translation of circRNAs (Zhang et al., 2018a), 41 differentially expressed circRNAs were found in GBM. The study was carried out using a RNA-seq approach coupled to a ribosomal RNA depletion so that any RNA without sequence alignment to the genome was extracted and processed to identify the circRNAs. Among these, circSHPRH was found to be significantly less expressed in GBM than in normal tissues. After confirming the capacity of circSHPRH to carry out 5′-cap-independent translation, the translation of **SHPRH-146aa** was validated by Western blot and LC-MS/MS-based with a unique peptide. In this study, the under-expression of SHPRH-146aa was also correlated with a lower patient overall survival. If the function of SHPRH-146aa was not fully elucidated, it was hypothesized that AltProt could prevent the RefProt SHPRH from degradation. More recently, Biswas et al. aimed at characterizing the structure of SHRPH-146aa to identify its interaction partners and the related signaling pathways (Biswas et al., 2021). Among the identified partners is DTL, an E3 ligase, involved in the response to DNA damage and its repair, as well as in the ubiquitination signaling. DTL was found to bind to the homologous sequence part between the AltProt SHRPH-146aa and its RefProt SHRPH, supporting the idea of a rescue function.

**FBXW7-185aa** is also an AltProt encoded from a circRNA (circFBXW7) which is described to be involved in GBM (Yang et al., 2018). Observed as under-regulated in cancer cells, it is assumed to play a role in the regulation of the proliferation and the cell cycle acceleration. FBXW7-185aa is an antagonist to USP28 which induces a reduction in the half-life of the c-Myc oncogene. The expression of circFBXW7 is correlated with the survival of GBM patients; likewise, the abundance of FBXW7-185aa is reduced in tumor regions compared to adjacent healthy regions. In GBM, the RTK/PI3K pathway is described as overactivated, supposedly through the activation of the serine–threonine kinase AKT (70% of GBM patients), then leading to proliferation and survival of tumor cells. The AKT family is divided into three isoforms, namely, AKT1, AKT2, and AKT3, but only AKT3 is described as mandatory for the transformation of astrocytes into glioma cells. If the importance of AKT3 was reported in GBM, none had been reported until 2019 about the importance of circAKT3 encoded on the same gene (Xia et al., 2019). CircAKT3 translates into an AltProt, **AKT3-147aa,** which has the opposite function to AKT3 since it activates PDK1 and inhibits the proliferation and the tumorigenicity of GBM cells and is found to be under-regulated in GBM tissues (Wu et al., 2020).

An 87-amino acid protein (**PINT-87aa**) issued from the circular RNA p53-induced transcript exon2 (circPINT/circPINTexon2) was also found in GBM by Ribo-seq and was both confirmed by MS and immunoblotting (Zhang et al., 2018). Identified in the nuclei of the cells, PINT-87aa regulates the RNA elongation of several oncogenes, in particular PAF1 (polymerase II-associated factor 1), an essential factor of transcriptional elongation, as well as CPEB1, SOX-2, c-Myc, and cyclin D1. In GBM, PINT-87aa is characterized by an under-expression in tumor tissues compared to the nontumor adjacent ones. Interestingly, a similar variation is also found in other cancers like breast cancer and gastric cancer. Besides, PINT-87aa knockout in xenograft shows a rapid increase in tumor volume, which suggests an antitumor effect of PINT-87aa.

More recently, **SMO-193aa**, a transmembrane AltProt, coming from the circular G protein-coupled-like receptor smoothened (circSMO), was correlated with survival in GBM. Indeed, an increase in SMO-193aa expression correlates with a poorer prognostic survival of patients. On the contrary, the inhibition of SMO-193aa expression suppresses the proliferation and tumor growth. SMO-193aa is involved in the shh/Glil/FUS expression loop involving the Hedgehog signaling activation (Wu et al., 2021).

Since circRNAs have only been described for a few years and their function remains still poorly understood, there is still a lot of questioning around their ability to sponge RNA and their ability to be translated into proteins. In GBM, these proteins seem to play a role in signaling pathways essential for tumor development through their direct interaction with oncogenic products, RNA, and proteins. circRNAs demonstrate once again that the genomic, transcriptomic, and proteomic landscape is much more complex than expected and that this complexity must be considered to the benefit of the discovery of new diagnostic and prognostic biomarkers and new therapeutic targets and to better understand the course of the pathology.

### An Alternative Vision of Proteomics in GBM

The translation of circRNAs into proteins has been demonstrated using Ribo-seq, RNC-seq, and RNA-seq which identify the binding of the transcript by the ribosome and the production of the translated nascent chain of proteins. Nevertheless, MS analysis coupled with large-scale interrogation from predicted databases makes it possible to identify a much larger number of new proteins from complex media.

In GBM, several large-scale studies of ghost proteins were conducted using MS-based proteomic approaches. A first study, conducted from cell lines and dedicated to the optimization of extraction and enrichment methods to improve AltProt detection, showed the detection of 89 different AltProts representing an average of 1% of total identifications but 70% of the total proteins with a <15-kDa molecular weight (MW). Interestingly, out of these 89 AltProts, only five were issued from mRNA, the rest being encoded from ncRNA 84 (Cardon et al., 2020b). If large-scale strategies lead to the identification of a high number of AltProts, accessing their functions is not straightforward and rather complex. However, the combination of gene enrichment, gene ontology, and the identification of protein–protein interactions (PPI) has demonstrated to help decipher the function of these proteins and to be applicable to dynamic studies of physiopathological mechanisms (Cardon et al., 2020a).

The search for PPI by cross-linking mass spectrometry (XL-MS) in the GBM cancer cells NCH82 showed the regulation of 81 AltProts under the stimulation of a factor activating the epithelial-to-mesenchymal transition (EMT). Among these 81 AltProts, a large number are from lncRNA. One AltProt (**AltLOC101927356**) translated from the transcript LOC101927356, which is specifically identified in the activated EMT condition, interacts with the proteins of the tropomyosin cytoskeleton (TPM), highlighting the relation of the protein to the cytoskeleton and potential function associated with cell morphology. On the other hand, the **AltLINC00624** protein derived from the ncRNA LINC00624 was found to potentially play a role in the regulation of protein expression which is in line with previous findings on proteins encoded from ncRNAs. In another proteomic study of epigenetic modifications (histone methylation) induced by the tumor on tumor-associated macrophages (TAMs), 10 AltProts have been identified in TAMs as significantly involved in the epigenetic modulation. Among the AltProts identified, there is **IP_1295370** encoded by the ncRNA AC106932.1 (Cardon et al., 2020c). Further work is needed to characterize its function; however, for the first time AltProt translated from ncRNA has been associated with epigenetics. Moreover, the study of AltProts in extracellular vesicles (EVs) produced by rat GBM cells identifies the presence of an AltProt **IP_2659453** translated from the ncRNA LOC103695286 (Murgoci et al., 2020). This AltProt which is involved in the communication of glioma cells may play a role in the proliferation and the cell. The application of large-scale MS-based methods is much more versatile than targeted strategies. They can be easily used to search data present in the public repositories or implemented in commonly used proteomic workflows.

**FIGURE 4:**
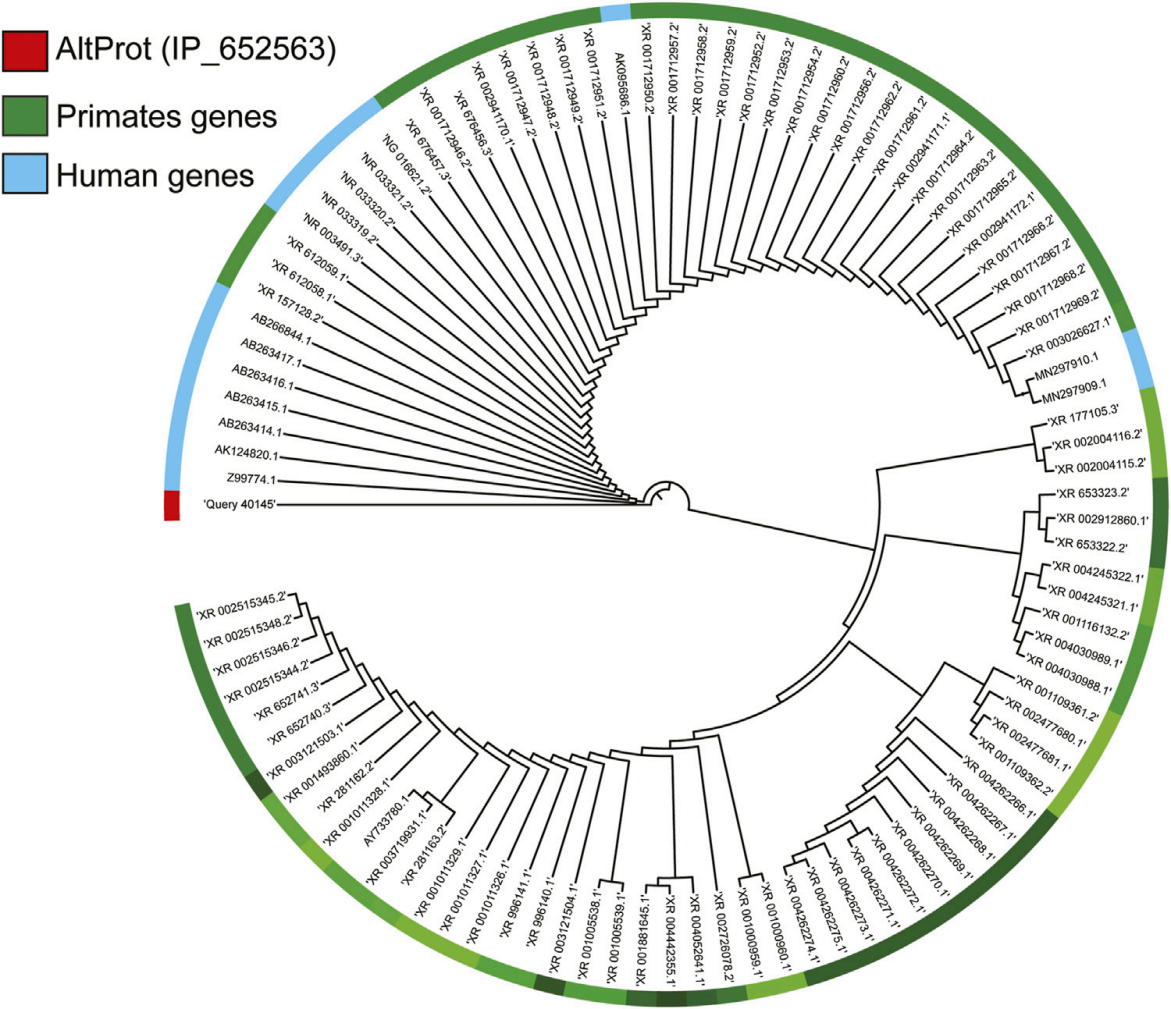
Study of the phylogeny of AltProt (IP_652563 identified in the clinical study of GBM). Based on its sequence homology with the known and predicted proteome of all the mammalian species present on BlastP, the AltProt sequence is only found in primates. In blue, the nearby human genes that can encode this sequence, and in green, those of other primates.

In 2017, an exploratory work aiming at grade III glioma classification and based on spatially resolved proteomics guided by mass spectrometry imaging (MSI) led to identification of 22 AltProts (Le Rhun et al., 2017) specific to tumor. Three are derived from ncRNAs, the **IP_204724** protein encoded from the ncRNA LOC221122, the **IP_149055** protein translated from the ncRNA TCF21 antisense RNA-inducing promoter demethylation (TARID), which is described to play a role in the regulation of demethylation by interacting with TCF21 and GADD45A, and the **IP_148329** protein from the noncoding HLA complex group 18 (HCG18) transcript, an ncRNA involved in tumor development *via* the modulation of miR-140/CCND1 and the Hedgehog signaling pathway. More recently, the combined MS imaging and spatially resolved proteomics strategy were applied on a GBM prospective cohort of 50 patients. Among all the identified proteins, 58 AltProts were found to be regulated in GBM of which 33 are translated from ncRNA (Drelich et al., 2020). One of these AltProts **(IP_652563)** has been correlated with the patient overall survival, and its expression is related to poorer survival. **IP_652563** is translated from the ncRNA Z99774.1. Very interestingly, this transcript might not be a fixed allele in the entire population, making it a major predisposition marker if correlated with a lower survival rate of GBM patients. This gene would be a *de novo* gene encoding a protein derived from an ancestral noncoding transcript. The phylogenetic alignment of the gene shows a strong homology only with primate (Figure 4), a sign of a recent appearance of this gene during the evolution. Notably, among the identified AltProts, 13 are translated from eight ncRNAs previously described in GBM as significant variants (RMST, HMMR-AS1, HOTAIR, MALAT1, ST7-AS1, DDX11-AS1, LBX2-AS1, RNF219-AS1). From these eight ncRNAs found in a previous study, 100 AltProts are predictable though 13 have been found in the reanalysis of the MS data, of which 11 are also present in the Ribo-Seq data used to cross-validate the OpenProt database (Table 1).

**TABLE 1:** Prediction of AltProt from ncRNA described in the literature involved in the pathology of GBM. The predicted AltProts are characterized by the detection of their peptides in the mass spectrometry data (MS detection) and their detection in the Ribo-seq data (translation detection), as well as the description of the known domains with the focus on the regions described to be the transmembrane (membrane domain).

ncRNA	Number of predicted AltProt	MS detection	Translation detection	Membrane domain
RMST	23	5	0	3
HMMR-AS1	5	0	0	2
HOTAIR	10	0	4	0
MALAT1	24	4	5	8
ST7-AS1	3	0	0	1 signal peptide
DDX11-AS1	7	1	2	1
LBX2-AS1	7	0	3	1
RNF219-AS1	21	3	0	2

AltProts which have remained hidden until recently are now revealed by proteogenomic strategies and, as supported by all these preliminary works, will undoubtedly be one of the keys for better understanding GBM and finding new treatment leads. Notwithstanding, significant work remains to be achieved to get the regulation and mutations of these proteins.

### Mutations in AltProts: A Deep Impact

**FIGURE 5:**
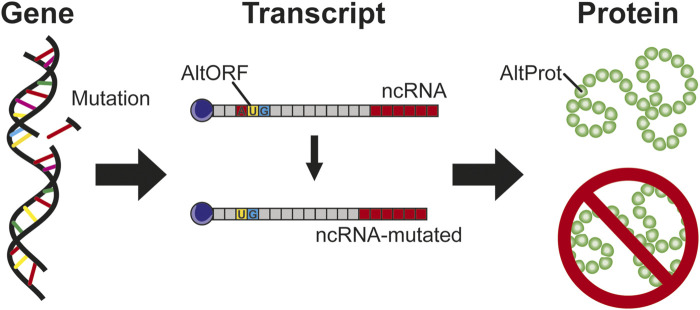
Gene mutations, if they impact region transcript in ncRNA, are considered as silent mutation without protein impact. In the case of AltProt expression, this kind of mutation can have a severe impact similar to the deletion of a start codon leading to the inhibition of the AltProt expression.

A key question is to determine if AltProts carry mutations as well as the impact of these mutations. In 2021, Erady et al. have predicted posttranscriptional modifications (PTMs) and mutations on AltProts in cancer, thanks to the combination of genome-wide association (GWAS), variants, and mutations in the Catalogue of Somatic Mutations in Cancer (COSMIC), Human Gene Mutation database (HGMD), and AltProt databases (Erady et al., 2021). Some of these mutations are described as impacting the regulatory region of genes implicated in cancer. However, the consequences of these mutations on the cell function and the impact on the pathology have not been described yet and are only explored in very few studies (Figure 5). However, as an example, it has recently been shown in endometrial cancer the presence of a mutation (C > T, COSM8898738) on the transcript encoding the RefProt GNL1 which has been described as a synonymous mutation with a silent impact on the protein. However, in the reading frame translating the AltProt AltGNL1, this mutation causes a premature stop codon (Cardon et al., 2021b). If this truncated proteoform has not yet been demonstrated, nor correlated with the pathology, such an impact on a protein must likely have consequences on its signaling pathway.

In GBM, in correlation with the data described above (Cardon et al., 2020a), AltMAP2 was found to be interacting with TPM4 in cancer cells. However, the transcript encoding this AltProt (ENST00000360351.8) bears 33 mutations which are localized in the region overlapping the coding DNA sequence (CDS) of MAP2 and the part encoding AltMAP2. Eight have been described to be silent mutations for the RefProts, although the analysis in the frame translating for the AltProt reveals that it is missense in that case (Table 2).

**TABLE 2:** Identification of mutations based on the COSMIC database for the transcript: ENST00000360351.8 coding for MAP2 RefProt. These mutations are considering silent on the MAP2 RefProt but with an impact on the sequence of the resulting AltProts.

CDS mutation	Legacy mutation ID	RefProt mutation	RefProt impact	AltProt mutation	AltProt impact
c.5304T > A	COSM8311680	p.A1768 =	Substitution - coding silent	p.L2Q	Substitution - missense
c.5331G > A	COSM461182	p.E1777 =	Substitution - coding silent	p.R11K	Substitution - missense
c.5340A > G	COSM7721686	p.T1780 =	Substitution - coding silent	p.H14R	Substitution - missense
c.5361C > T	COSM4700229	p.S1787 =	Substitution - coding silent	p.A21V	Substitution - missense
c.5394C > T	COSM7948190	p.S1798 =	Substitution - coding silent	p.P32L	Substitution - missense
c.5397G > A	COSM4807131	p.S1799 =	Substitution - coding silent	p.R33H	Substitution - missense
c.5409C > T	COSM7110613	p.I1803 =	Substitution - coding silent	p.S37L	Substitution - missense
c.5475G > A	COSM4552025	p.Q1825 =	Substitution - coding silent	p.R59K	Substitution - missense

Likewise, the mutations carried by the ncRNAs are considered to be silent because no proteins are expected to be translated from these RNAs. However, the AltProts resulting from these ncRNAs will necessarily be impacted by these mutations. For instance, from the MALAT1 transcript (accession: ENST00000534336.1) (Gutschner et al., 2013), 19 AltProts are predicted. In addition, in the TCGA mutation database, this transcript is described to be mutated for GBM (CPTAC-3 project) in squamous cell neoplasms, adenomas, and adenocarcinomas. Among the 61 enumerated mutations, 21 are found in regions from which an AltProt translation can be expected. Overall, more than 30% of mutations are expected for AltProts and could impact the ghost proteome, previously unconsidered because on ncRNAs which were assumed not to translate into any protein, the existence of AltProts has changed the game and makes them important to take into account (Table 3). Moreover, less than 10% (2/21) of these mutations will not change the amino acid sequence of the translated AltProts and be silent. Among the remaining 90% of mutations which will affect the amino acid sequence, ∼40% (9/21) will be missenses, ∼10% (2/21) will be in frame deletions, and ∼40% (8/21) will induce a frameshift leading to a radical change in the sequence including even the appearance of a premature stop codon changing the size of the translated protein. From the CPTAC-3 project in GBM, interestingly five mutations are identified to have a major impact on four different AltProts with amino acid sequence modifications (IP_204,844, IP_204,852, IP_204,854, and IP_204,839): one missense (1/4 of the AltProt) and three frameshifts with an early stop codon (3/4 of the AltProt). The GBM cohort of the CPTAC-3 study only registers five patients. However each of these mutations was identified at least in one patient, thus representing 20% of the mutations in GBM. The other 16 mutations impacting AltProts are all identified in adenomas and adenocarcinomas. When extending the analysis to all the TCGA projects referring exclusively to glioma, 37 mutations are found to impact MALAT1. Among these 37, 12 impact AltProts (including the four identified in CPTAC-3) and only 2/12 mutations are silent, the 10 other mutations leading to a change in the AltProt amino acid sequence (4 frameshifts, four missenses, two coding for an early stop codon). In this larger cohort of 53 patients, these mutations represent a rate of 1.89%.

**TABLE 3:** Demonstration of the mutations (TCGA database) impacting the ncRNA MALAT1 in the GBM and the impact that these mutations may have on the AltProts predicted as resulting from this transcript, described as having a specific variation in the GBM.

Chromosome location	CDS mutation	Type	AltProt	AltProt mutation	Type
65501319	delAG	Deletion	IP_204854	23.RKQENPISG > KTRKSNIRI	Frameshift
65501319	delAG	Deletion	IP_204839	7.ENKKIQYQ > KQENPISG*	Frameshift
65498351	delTG	Deletion	IP_204844	32.LGGRRSEWATGSQRPPGLRRGAALWCG > RGPQIRVGHWQPTAPGAQAGSSSVVWD*	Frameshift
65501093	delTG	Deletion	IP_204852	18.VCLVGVMKYFSFV > MFSWGNEVFQFCE*	Frameshift
65501435	C > A	Substitution	IP_204839	A55D	Missense
65503129	T > C	Substitution	IP_204840	S28P	Missense
65498455	T > G	Substitution	IP_204844	F67C	Missense
65506086	T > C	Substitution	IP_204846	L28P	Missense
65501428	G > T	Substitution	IP_204839	E43*	Stop Gained
65503714	delTC	Deletion	IP_204855	S31*	Stop Gained
65503128	T > C	Substitution	IP_204840	=	Synonymous
65498330	C > T	Substitution	IP_204844	=	Synonymous

These exploratory results issued from predictions shed light on the potential impact of mutations on the ghost proteome. Nonetheless, they again highlight the importance of considering such mutations in future studies. These mutations directly affect the nucleic acid sequence of the ncRNAs and consequently can also affect their function through the sequence modifications (e.g., conformational changes). Besides, the mutations of the ncRNA will potentially cause various changes to the AltProt level such as translation inhibition, translation of truncated AltProt proteoforms, or amino acid sequence modifications all with the potential to deeply affect the functions of these proteins.

## Discussion

Because the unveiling of the ghost proteome has shaken up a well-established paradigm, AltProts are always considered with caution despite the various studies pointing to the existence of these novel proteins and their importance in different pathologies. The polycistronism in eukaryotes could finally be perceived as a sign of evolution, such as the previously discussed IP_652563 protein, which would be a *de novo* gene encoding a protein derived from an ancestral noncoding transcript. Interestingly, some AltProts are also conserved across different species (Samandi et al., 2017). The polycistronism mechanism is also observed in the viral infection and some viruses which have all the coding information for their protein synthesis contained in a single RNA (Fouillot et al., 1993; Suzuki et al., 1996; Li, 2019). Once infected, the cell starts the translation of the viral proteins despite those being encoded from a single RNA containing multiple ORFs. Furthermore, the recent demonstration of alternative translation paths such as the IRES-mediated translation and the initiation of m6A-dependent translation on the circRNAs is derived from the description of viral mechanisms (Kong et al., 2020). The presence of one site allowing the binding of ribosomes in noncoding regions adds further weight to the existence of AltProts and the ghost proteome. We expect even more proteins to be translated through IRES and m6A on ncRNAs and on noncoding regions of mRNAs (Mouilleron et al., 2016). As for the function of these AltProts, a lot of work remains to be done and many discoveries to be made.

The preliminary work conducted to get insight into these novel proteome landscapes has demonstrated their implication in critical signaling pathways, such as the regulation of translation and gene expression, with a specific role according to the physiopathological context. In 2014, the Toddler peptide, which is an AltProt resulting from the translation of an ncRNA, was identified during the embryonic formation of the zebrafish and is coupled to a G protein receptor, thus interacting to an orphan G protein receptor activator (Pauli et al., 2014). This AltProt once again shows the diversity of functions carried by these proteins. Recently, AltProts issued from noncoding and frameshift regions were found to be neoantigens and presented by MHC-I (Ruiz Cuevas et al., 2021). Among the predicted AltProts issued from MALAT1 (Table.3) together with their listed mutations, it is possible to predict the production of an immunogenic peptide (NetMHCpan4.1) from IP_204,839. Besides, two immunogenic peptides can be predicted to bind the HLA-A01:01 (VTENKKIQY-Strong binding and SIGEMAGSY-Weak Binding). In addition, the chr11: g.65501435C > A (TCGA) mutation causes an amino acid sequence modification on the A55D AltProt that could lead to the translation of a specific peptide presented by HLA-A: EMDGSYSFF described as weak binding. These peptides which are essential to the establishment of the inflammatory response represent interesting new diagnostic/therapeutic targets that require further investigations. Also important to note is that a significant number of the predicted AltProts have a transmembrane domain, some presenting a signal peptide and an extracellular domain indicating a possible extracellular secretion (Brunet et al., 2020) (see Table.1). In addition, not only the AltProts have to be considered but also the mutations they could carry. Several mutations described as silent for the RefProts are not silent for the relative AltProts. Sometimes, these mutations can also impact both RefProt and the AltProt, resulting in an additional level of complexity with a pathological impact currently only attributed to RefProt, while the impact of AltProt is there as well but remains to be deciphered.

Every new advance in the understanding of the cell functions brings new answers but also more questions, such as for example the recent existence of extracellular ribosomes that have been detected outside the cell (Tosar et al., 2020). These ribosomes are actually coupled to RNAs, and even if this seems improbable, there might be at the origin of an extracellular translation, which could regulate differently and independently from cellular proteins. This again demonstrates that the world of protein translation is highly complex and has not yet been fully elucidated. While the study of AltProts unveils some part of the hidden proteome, there is probably still more to be discovered.

**FIGURE 6:**
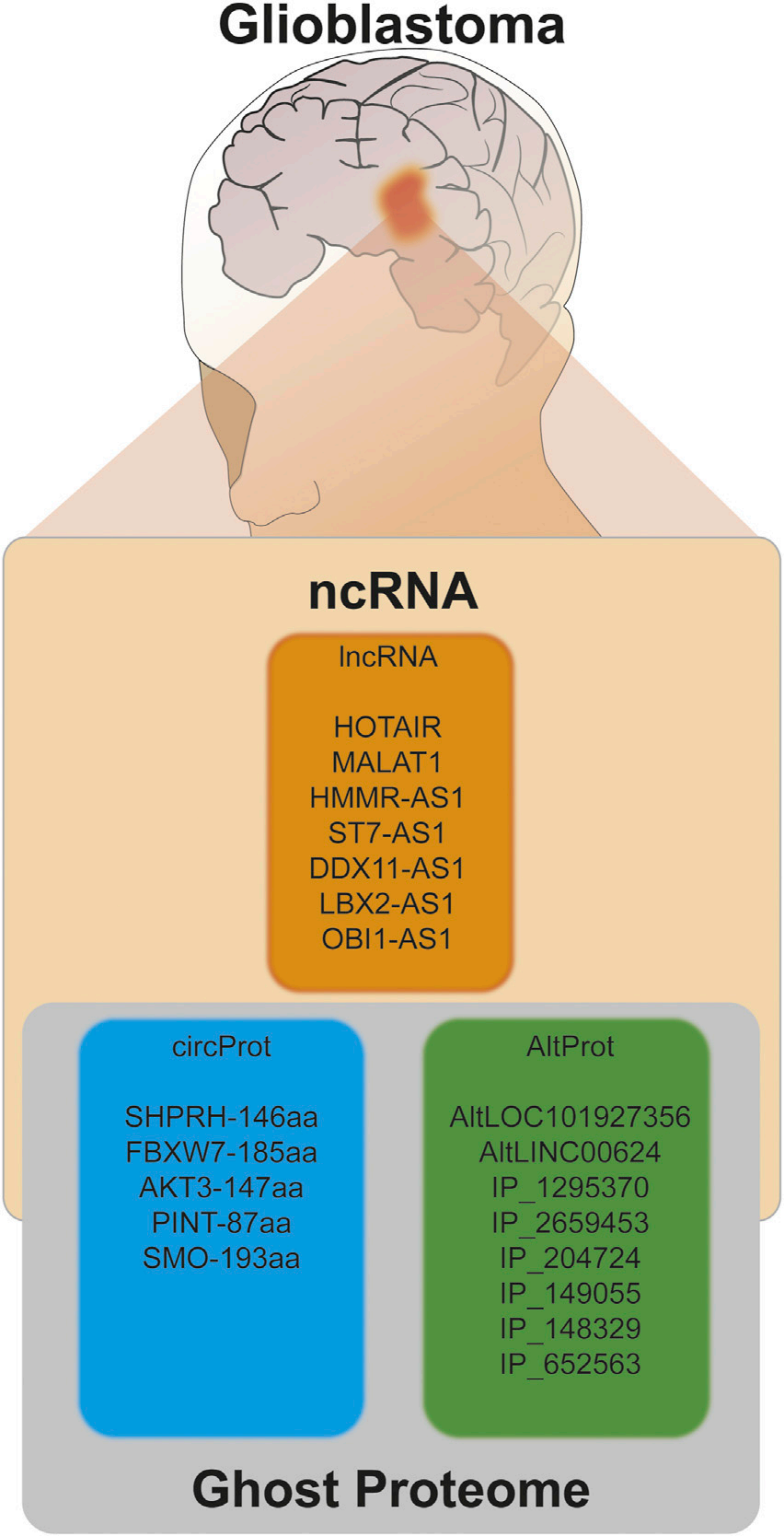
Summary of different hidden players that are involved in the pathology of glioma.

In our work, we consider the GBM study a fertile ground for assessing this ghost proteome, as presented in this review and illustrated in the presentation of the different RNAs (circRNA, lncRNA), their AltProt products and the techniques developed for their identification making possible to demonstrate their significant variation in the pathology (Figure 6). The existence of IRES and m6A modification for translation which are triggered during cellular stress correlate with tumor development and excessive proliferation of cells. Uncovering these proteins in GBM opens the door to a new hope for early detection of the pathology and of these different grades, the stratification of the patients, and the access to new therapeutic targets which may one day make it possible to better treat GBM and improve patient overall survival.

## Nomenclature

AltProt: alternative Proteins
circRNA: circular RNA
COSMIC: Catalogue of Somatic Mutations in Cancer
DDX11-AS1: DEAD box 11 antisense RNA1
EMT: epithelial mesenchymal transition
ENCODE: Encyclopedia of DNA elements
endo-siRNA: Endogenous short interfering RNAs
GBM: Glioblastoma
GWAS: genome-wide association
HGMD: Human Gene Mutation database
HMMR-AS1: Hyaluronan-mediated motility receptor antisense RNA1
HOTAIR: HOX Antisense Intergenic RNA
HRMS: high resolution mass spectrometry
IDH: isocitrate dehydrogenase
IRES: internal ribosome entry site
ITAF: IRES transacting factor
LBX2-AS1: ladybird homeobox 2 antisense RNA1
lncRNA: long non-coding RNA
m6A: methylation of the N6 group position of the adenosine
MALAT1: Metastasis associated lung adenocarcinoma transcript 1
miPEP: micropeptides
miPEPs: miRNA-encoded peptides
miRNA: microRNAs
MSI: mass spectrometry imaging
ncRNA: non-coding ribonucleic acid
OBI1-AS1: ORC ubiquitin ligase 1 antisense RNA 1
ORF: Open Reading Frame
piRNA: Piwi-interacting RNA
POU4F1-AS1: POU class 4 homeobox 1 antisense RNA 1
PPI: protein-protein interaction
PTMs: post translational modifications
RefProt: reference proteins
Ribo-seq: ribosome profiling
RNC-seq: ribosome-nascent chain complex sequencing
rRNA: ribosomal RNA
sdRNA: Self-deliverable siRNA
SEPs: sORF-encoded peptides
smORF: small Open Reading Frame
smProt: small or short proteins
sncRNA: short non-coding RNA
snoRNA: small nucleolar RNAs
snRNA: small nuclear RNA
TCGA: The Cancer Genome Atlas
tRNA: transfer RNA
WHO: World Health Organization
XL-MS: crosslink coupled to mass spectrometry
